# Reduction of surface charging effects in laser ablation ionisation mass spectrometry through gold coating

**DOI:** 10.1039/d3ja00078h

**Published:** 2023-05-23

**Authors:** Salome Gruchola, Andreas Riedo, Peter Keresztes Schmidt, Coenraad P. de Koning, Luca N. Knecht, Marek Tulej, Frances Westall, Peter Wurz

**Affiliations:** a Physics Institute, Space Research and Planetary Sciences, University of Bern Switzerland salome.gruchola@unibe.ch; b CNRS Centre de Biophysique Moléculaire Orléans France; c NCCR PlanetS, University of Bern Switzerland

## Abstract

In femtosecond Laser Ablation Ionisation Mass Spectrometry (fs-LIMS) short laser pulses are used to ablate, atomise, and ionise solid sample material shot-by-shot. When ablating non-conductive samples electric charging of the surface can occur. Depending on the geometry of the instrument, the surface charge can influence the spread of the ablation plume and reduce spectral quality. Methods to reduce surface charging were investigated using a non-conductive geological sample and a miniature fs-LIMS system with a co-linear ablation geometry. Pausing five seconds between consecutive laser bursts fired on non-coated material improved the spectral quality by giving surface charges more time to dissipate. However, best mass spectrometric results were achieved after the sample was sputter coated with a thin gold layer, as a conductive sample surface hinders charge build-up. Consequently, gold coating allowed operation of the laser system at much higher laser pulse energies improving sensitivity and reliability. It also removed the need to pause between laser bursts, speeding up the measurement acquisition.

## Abstract 2

In this study, two methods are investigated to reduce the adverse effects on spectral quality that can occur when investigating non-conductive samples with a miniature laser ablation ionisation mass spectrometer.

## Graphical abstract

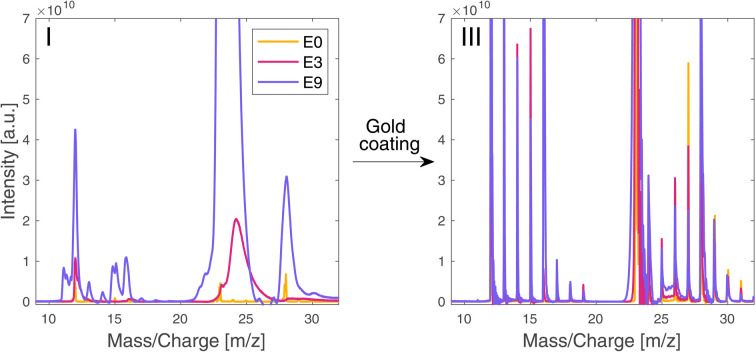

## Introduction

In laser ablation ionisation mass spectrometry (LIMS), laser pulses are used to ablate material from the solid sample surface, with simultaneous partial atomisation and ionisation of the removed material. This produces an ablation plume with a mix of atoms, polyatomic species, their ions, and electrons. As a consequence of heating the irradiated surface, its thermal expansion, and charge separation, positively charged ions are ejected from the surface *via* Coulomb explosion and accelerated by the electric fields of the mass analyser, whereas the ejected electrons are pushed back onto the surface.^1^ This results in a local charge build-up on non-conductive samples.^2–4^ Surface charging affects laser ablation ionisation instruments where the plasma is guided into the system's mass analyser directly after ablation. The electric field of the surface charge influences the ion spread, which often results in reduced spectral quality. Surface charging is also a common problem in Scanning Electron Microscopy (SEM), and the standard technique to reduce it is to sputter coat non-conductive samples with a thin conductive layer, usually with high-purity gold or carbon in the case of geological samples.^5,6^ Gold coating of non-conductive samples has also been applied in Secondary Ion Mass Spectrometry (SIMS),^7^ extreme ultraviolet laser ablation ionisation mass spectrometry (EUV-TOF-MS)^8^ and matrix-assisted laser desorption/ionisation tandem mass spectrometry (MALDI-TOF/TOF-MS).^9^ In this contribution, two techniques for reducing surface charging occurring when studying non-conductive samples with a fs-LIMS system were investigated; first, the time available for charge dissipation between laser shots was increased, and second, the sample was sputter coated with a thin gold layer.

## Experimental

### Instrument

**Fig. 1:**
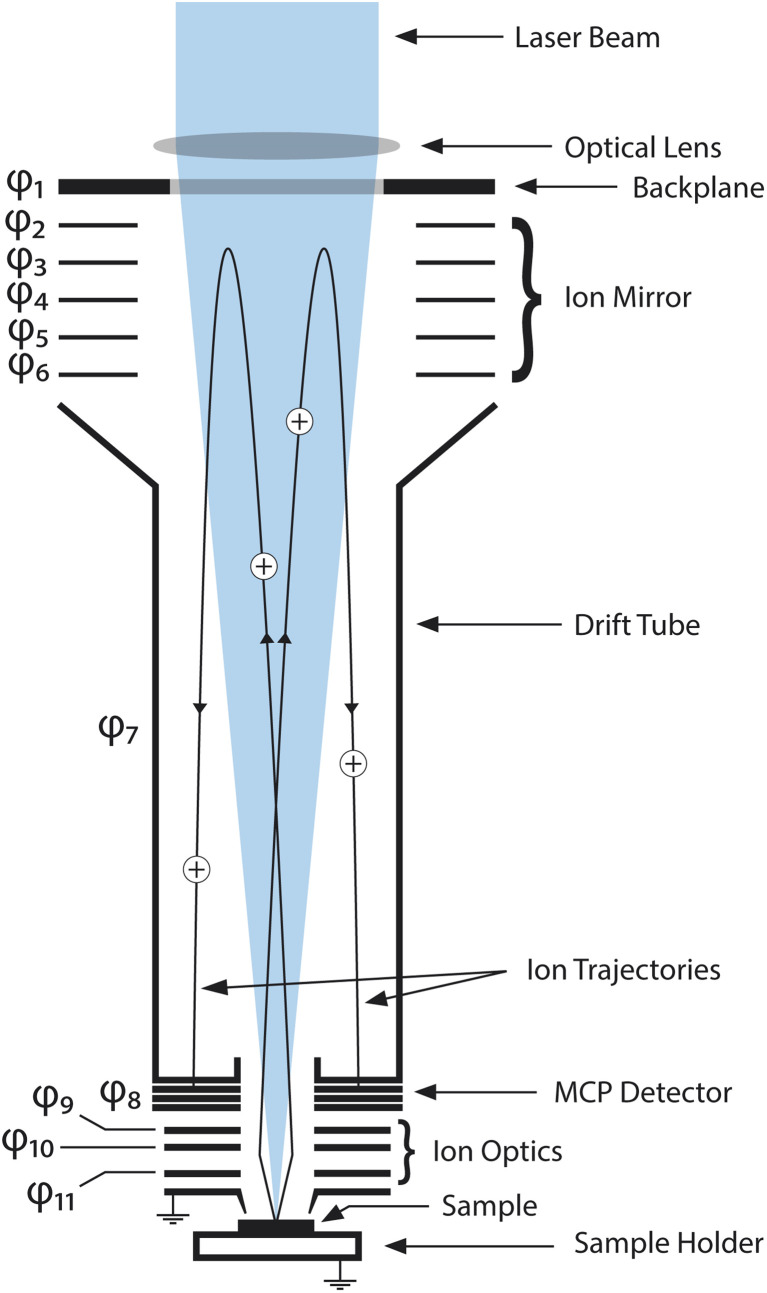
Schematic drawing of the LMS instrument mass analyser. The laser beam enters the mass analyser from the top by passing through a focusing lens and through the backplane. The sample is placed on a sample holder (at ground potential) and placed several hundred micrometers below the entrance electrode (at ground potential) in the laser beam focus. Ablated ions are accelerated and focused by the ion optics and led onto their trajectories through the mass analyser. After being reflected once by the ion mirror, the ions hit the MCP detector system and are recorded by the acquisition system of the instrument. Electrode potentials *φ*_1_–*φ*_11_ are summarised in Table 3 in the appendix.

The measurements in this study were conducted using a miniature laser ablation ionisation mass spectrometer (LIMS) built at the University of Bern. The instrument was originally designed for *in situ* analysis of the chemical composition of solids on surfaces of solar system bodies. It is a compact reflectron-type time-of-flight mass spectrometer (dimensions of 160 mm × ∅ 60 mm), coupled to a femtosecond laser system (Ti:sapphire laser system, CPA-Series, Clark-MXR Inc., USA, with *λ* = 258 nm, *f* = 1 kHz, τ ∼180 fs pulse duration) achieving lateral spatial resolutions of ∼10 μm on the investigated sample surface. Once generated during the laser ablation process, ions are guided through the mass analyser by the ion optical system and recorded by a multichannel plate (MCP) detector system with a high-speed acquisition system (12 bit vertical resolution, 3.2 GS s^−1^ sampling rate). A schematic drawing of the mass analyser can be found in Fig. 1. Data analysis is conducted with an in-house written software suite.^10^

The instrument design and measurement principle are described in detail in previous publications.^3,4,11–16^

The occurrence of surface charging depends on many parameters, including laser power density, the interaction of the sample material with the laser radiation, the size of the sample, the mounting of the sample on the sample holder and the voltages applied to the ion optics of the mass analyser. This explains why in the past numerous studies on non-conductive samples were conducted successfully with our LIMS instrument.^17–23^ For certain samples, however, surface charging might have limited the maximally possible laser pulse energy and hence the detectability of elements and isotopes at trace level abundances.

### Materials and methods

The non-conductive sample chosen for the study is a 30 μm thin section of a 3.33-billion-year-old (Ga) rock (chert) from the Barberton Greenstone Belt, South Africa. It is mounted on a standard glass slide and fixed to a stainless-steel sample holder with conductive copper tape. Previous measurements on the sample yielded overall well resolved spectra within normal operating performance with a low number of bad quality spectra (<1‰), for which mass unit resolution was not achieved in the range of 1–250 amu, meaning that at least one pair of mass peaks with a mass difference of ∼1 amu could not be resolved. However, the laser pulse energy was too low to detect certain trace elements, and MCP potentials could not be increased without saturating the detector. Because of the massive early diagenetic silicification of the sample (now > 98% SiO_2_) interesting trace elements are at lower end of the limit of detection of the used instrument (lower μg g^−1^ range for masses in the range of 1–250 amu). In general, for inhomogeneous samples, as *e.g.*, geological samples containing different minerals, it can be challenging to find a single laser pulse energy, which yields good spectral quality over the whole studied area while also operating with a sufficiently high sensitivity. For the present sample, the spectral quality degraded significantly at energy levels where detections of depleted trace elements might have become possible.

As surface charging has so far not been of concern for conductive samples, the sample was gold coated using a standard sputter coater (SCD 005, BAL-TEC GmbH, Switzerland) usually used for coating of non-conductive samples prior to SEM analysis. A sputter time of 50 s at a current of 50 mA and at a sputter distance of 5 cm was used. According to the specifications of the sputter coater, the resulting gold layer had a thickness of approximately 12 nm. The exact thickness of the gold layer and the homogeneity of the gold distribution is of less importance for this study than it would be for SEM, as the only objective was the creation of a conductive sample surface. The resistance between the sample surface and grounded sample holder decreased from > 40 MΩ before gold coating to ∼16 Ω after gold coating (measured with a digital multimeter Fluke 85III). Apart from sputter coating the sample, no further sample preparation step was conducted.

It is possible that in future LIMS studies the natural gold abundance of a samples is of interest. In that case, gold sputter coating would no longer be an option and other species would need to be used for the conductive coating such as palladium, platinum or carbon.^5,24^

The spectral quality was studied before and after gold coating the sample by conducting energy campaigns with 10 different laser pulse energies between 0.5 μJ and 2.75 μJ, in steps of 0.25 μJ (see Table 1). With laser ablation crater diameters of ∼10 μm, laser pulse durations of 180 fs and a laser energy transmission of 28% from the point of measurement to the sample surface, the estimated laser power densities are in the range of 9.9 × 10^11^ W cm^−2^ to 5.4 × 10^12^ W cm^−2^ for energy levels E0 to E9. The laser power density at E18 equals approximately 9.9 × 10^12^ W cm^−2^. Laser shots were applied in bursts of 200 single laser shots and a total of 25 bursts per laser pulse energy were fired on each surface location. The data from the first 5 bursts were excluded from the subsequent analysis due to the crater formation processes.^25^ The laser system has an intra-burst repetition rate (IBRR) of 1 kHz, and a default burst repetition rate (BRR) of 40 Hz for bursts of one single laser shot. The BRR decreases for larger numbers of single laser shots per burst. Before and after gold-coating, the energy campaigns were conducted at a default BRR of 4.4 Hz, (highest repetition rate possible for bursts of 200 single laser shots), and additionally at a lower BRR of 0.2 Hz (corresponding to a 5 s pause between laser bursts). At the lower BRR, the surface charge has more time to dissipate. The IBRR remained unchanged at 1 kHz throughout all measurement campaigns. In the following, the four measurement campaigns are labelled MC I–MC IV (for description see Table 2). For each energy level E0–E9 of each measurement campaign a new location was selected on the sample. In total, 40 locations were studied in a grid of 4 × 10 craters, with a pitch of 25 μm between craters.

**Table 1:** Laser pulse energies used for the pulse energy campaigns

	Pulse energy [μJ]		Pulse energy [μJ]
E0	0.49 ± 0.05	E6	2.06 ± 0.05
E1	0.76 ± 0.05	E7	2.27 ± 0.05
E2	1.01 ± 0.05	E8	2.53 ± 0.05
E3	1.27 ± 0.05	E9	2.75 ± 0.05
E4	1.55 ± 0.05	E18^a^	5.01 ± 0.06
E5	1.78 ± 0.05		

a Only used on the gold coated sample.

**Table 2:** Summary of the measurement campaigns (MCs) conducted on the sample before and after gold coating. BRR stands for burst repetition rate

MC	Gold coated	BRR [Hz]	Acquisition time
I	No	4.4	∼9 min
II	No	0.2	∼3.6 h
III	Yes	4.4	∼9 min
IV	Yes	0.2	∼3.6 h

To investigate the spectral quality quantitatively, the three parameters ion yield (integrated peak areas), mass resolution (*m*/Δ*m*) and signal-to-noise-ratio (SNR, ratio of maximum peak intensity to standard deviation of noise floor) were determined and compared for the different MCs and all elements detected with an SNR > 5.

## Results and discussion

### Spectral quality

**Fig. 2:**
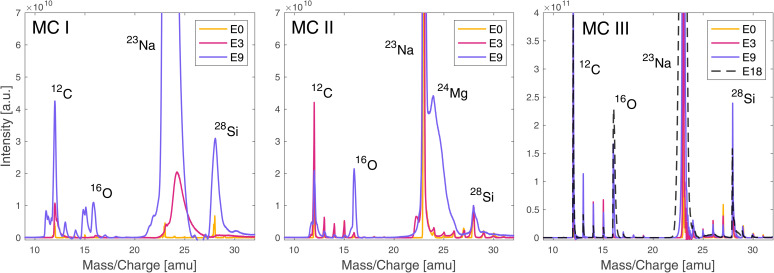
Mass spectra recorded before (MC I, MC II) and after gold coating the sample (MC III), without (MC I, MC III) and with (MC II) pausing for 5 s between the laser bursts. Spectra are shown for the three different laser pulse energies E0, E3 and E9 (Table 1). The figure showing the measurements after the gold coating (MC III) also displays the spectrum recorded at laser pulse energy E18 (Table 1). Each spectrum is the mass calibrated average of all TOF spectra recorded in bursts 6–25 at the given laser pulse energy. For a summary of the measurement parameters see Table 2.

The mass spectra obtained from the laser irradiance campaigns before and after the gold coating the sample surface are summarised in Fig. 2. For the description of the measurement campaigns (MCs) please see Table 2. MC IV is not shown in Fig. 2 as the spectral quality did not differ visibly from MC III.

As can be observed in Fig. 2, the spectral quality is slightly better for MC II compared to MC I, because of the 5 s delay between laser bursts that gives the sample surface more time to dissipate localised charge build-up. The improvement is not significant and comes at the cost of much longer acquisition times, hence measurement time, but has the advantage that no additional sample preparation step is required. Especially for *in situ* analyses or highly precious sample material where gold coating is not an option, this represents a mitigation strategy in case surface charging becomes a problem. After gold coating, the spectral quality improved significantly over the full investigated laser energy range (MC III). Even at the highest applied pulse energy possible (E18) no spectral quality degradation due to surface charging was observed; the spectra remained well resolved. Gold coating increased the spectral quality by increasing mass resolutions and improving the peak shapes, and enhanced the ion yields, which strongly indicates that the problem leading to low quality spectra is surface charging and not space charging within the ablation plume. If space charging would be the problem, a higher ion yield would decrease the spectral quality even more.

### Ion yield, mass resolution and SNR

**Fig. 3:**
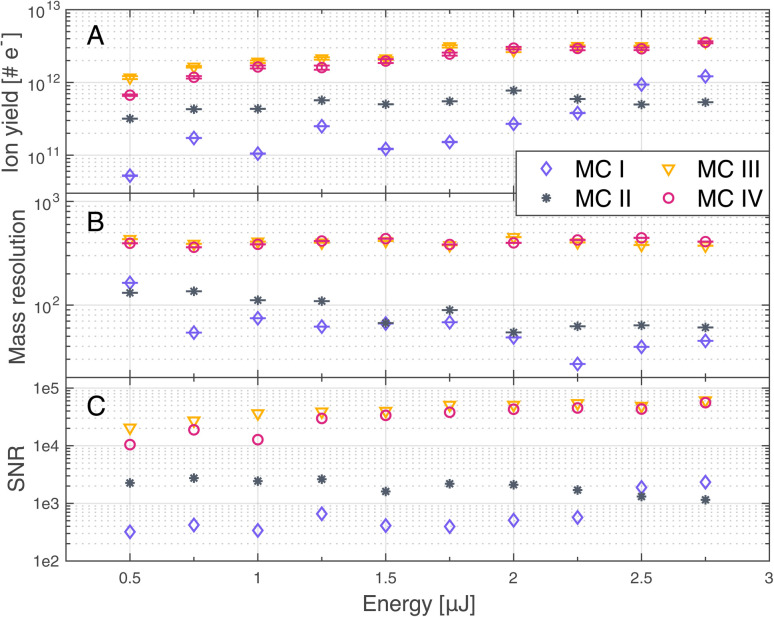
Ion yield (A), mass resolution (B) and signal-to-noise ratio (SNR, C) of ^12^C, measured at 10 different laser pulse energies. Measurements were conducted before (MC I, MC II) and after (MC III, MC IV) gold coating the sample, each at two different burst repetition rates (BRRs) of 0.2 Hz (MC II, MC IV) and 4.4 Hz (MC I, MC III), see Table 2.

A comparison of ion yields, SNRs, and mass resolutions are shown in Fig. 3 for ^12^C, in panels A, B and C, respectively. ^12^C was selected for this comparison as it was one of the most abundant elements in the studied region on the dark carbonaceous material, while the corresponding mass peaks remained resolved throughout all measurements. A certain variability of the carbon abundance across the studied area must be assumed, as no species are expected to have a constant abundance in the carbonaceous region. Measurements were conducted at 10 different laser pulse energies E0–E9 (see Table 1) in four different measurement campaigns (MCs I–IV, see Table 2). For the measurements conducted on the gold-coated sample (MC III and MC IV), the ion yield of the ^12^C peak increased with increasing laser pulse energy. For the measurement conducted before the gold coating (MC I and MC II), the ion yield also shows a trend towards higher values for higher laser pulse energies, however, the values fluctuate more. Especially MC I conducted before gold coating at a BRR of 4.4 Hz shows highly unstable results. Furthermore, the difference between the two measurements conducted before the gold coating (MC I and MC II) is more pronounced compared to the difference between measurements after gold coating (MC III and MC IV). At the lowest applied pulse energy, the difference in measured ion yields between MC II and MC III and MC IV is smaller than at highest energy, but still present, even though surface charging effects were minimal even on the uncoated sample. This shows that the gold coating does not only reduce surface charging but generally enhances ion yields. This can be attributed to a better laser–sample interaction or better plasma plume expansion towards the mass analyser.

Analogous to the ion yield of ^12^C, one can also see an increase of the SNRs (panel C) with increasing laser pulse energy on the gold coated sample (MC III and MC IV), whereas the SNRs rather tend to decrease on the uncoated sample (MC I and MC II). Again, the measurements conducted before gold coating are less stable (MC I and MC II), especially MC I, and the difference between the two campaigns is more pronounced compared to the two campaigns after gold coating (MC III and MC IV). Panel B shows that the mass resolutions tend to be higher with the gold coating than without the gold coating, which translates to higher spectral quality with gold coating.

Note that the results of MC III and MC IV are almost identical, which implies that the electrical conductivity of the gold coating is high enough for the surface charge to dissipate rapidly and pausing for five seconds between bursts brings no further improvement.

**Fig. 4:**
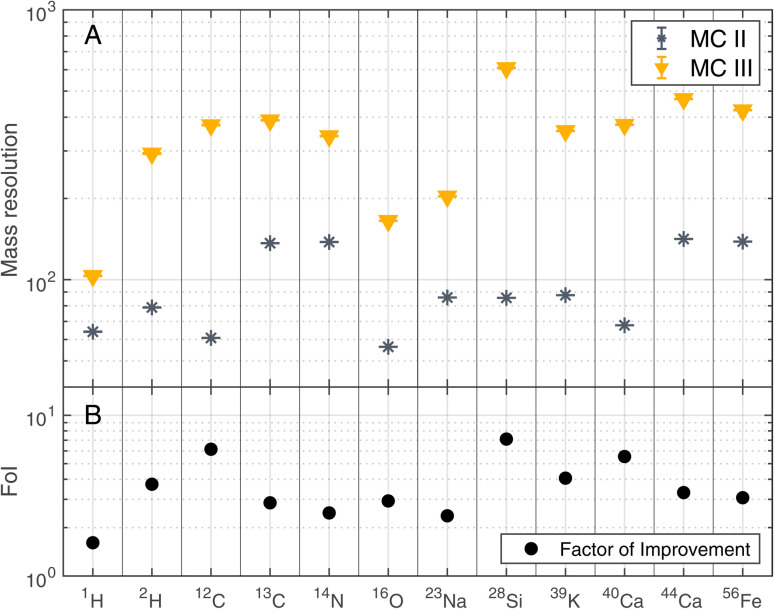
Mass resolution of selected masses recorded at laser pulse energy E9 (see Table 1) before (MC II) and after (MC III) gold coating the sample (panel A). BRRs of 0.2 Hz and 4.4 Hz were used for MC II and MC III, respectively. In panel B, the Factor of Improvement (FoI) is shown, which corresponds to the ratio between the mass resolutions measured after (MC III) and before the gold coating (MC II).

**Fig. 5:**
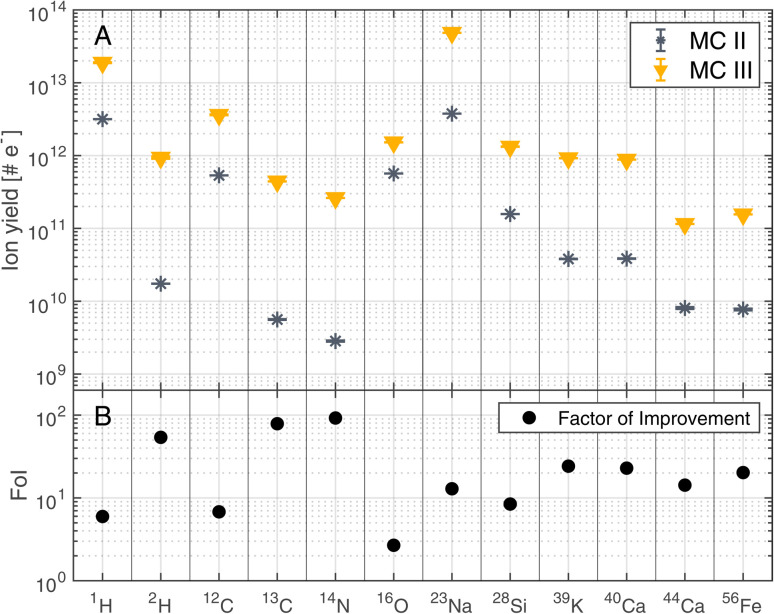
Ion yields of selected masses recorded at laser pulse energy E9 (see Table 1) before (MC II) and after (MC III) gold coating the sample (panel A). BRRs of 0.2 Hz and 4.4 Hz were used for MC II and MC III, respectively. In panel B, the Factor of Improvement (FoI) is shown, which corresponds to the ratio between the ion yields measured after (MC III) and before the gold coating (MC II).

**Fig. 6:**
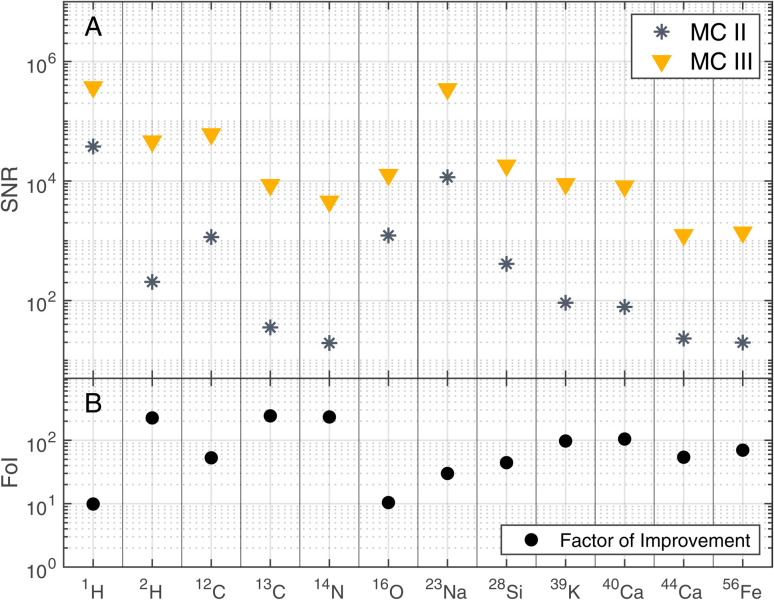
SNRs of selected masses recorded at laser pulse energy E9 (see Table 1) before (MC II) and after (MC III) gold coating the sample (panel A). BRRs of 0.2 Hz and 4.4 Hz were used for MC II and MC III, respectively. In panel B, the Factor of Improvement (FoI) is shown, which corresponds to the ratio between the SNRs measured after (MC III) and before the gold coating (MC II).

The improvements shown in Fig. 3 were not only observed for ^12^C but for other detected elements as well. Fig. 4 summarises the mass resolutions for various elements, both for metals and non-metals, including volatiles. The corresponding figures for the ion yields and the SNRs can be found in the Appendix section (Fig. 5 and 6, respectively). Results are shown for the measurements conducted before and after gold coating the sample, at a BRR of 0.2 Hz and 4.4 Hz, respectively (MC II and MC III). The data shown were recorded at the laser pulse energy E9 (see Table 1), the highest energy used both before and after gold coating. Note that the mass peak observed at ^56^Fe likely has contributions both from ^56^Fe and the cluster ^28^Si_2_, as the mass resolution of the instrument is not high enough to resolve this isobaric interference. For all analysed elements, the mass resolution was higher after the gold coating (panel A). The Factor of Improvement (FoI) is shown in panel B, corresponding to the ratio between the mass resolutions measured after and before gold coating. The maximally achieved FoI is around 7. In summary, gold coating makes it easier to obtain good quality spectra over the whole analysed area of a chemically inhomogeneous sample.

## Conclusion

We investigated two different methods to decrease the effects that surface charging can have on spectral quality when studying non-conductive samples with our fs-LIMS system. We looked at the effect that decreasing the laser burst repetition rate has by decreasing it from the default 4.4 Hz to 0.2 Hz (equivalent to a 5 s pause between subsequent bursts) and we sputter coated the sample with a thin gold layer to obtain a conductive sample surface. Both methods improved the spectral quality, with latter having the more significant effect, allowing for well resolved spectra up to the maximally possible laser pulse energy of ∼5 μJ of the used fs-laser system. Gold coating also enhanced ion yields and SNRs at the same pulse energy. Furthermore, without the need to pause for 5 s after each laser burst, the time for the acquisition of the data was reduced by a factor of 24, from ∼3.6 hours to ∼9 minutes only.

Gold coating of a sample does come at the cost of introducing an additional sample preparation step, whereas previously, no major sample preparation was applied prior to LIMS analysis. Furthermore, gold coating is currently not a feasible technique for space missions. However, methods like indium coating could possibly be developed seeing that indium has been used for micro-thrusters in space.^26,27^ Otherwise, it should always be possible to reduce the BRR. For all non-space related analyses for which LIMS will be used for in the future, it can be of high advantage to sputter coat non-conductive samples beforehand. Even more so for the laboratory scale version of our LIMS instrument.^28,29^ Especially, since the coating procedure itself is very fast (∼1 min for a 12 nm layer of gold). A future study to analyse in more detail how gold coating affects quantification would be of interest. Studies with different coating metals could also be considered, however, no fundamental differences are to be expected if the conductivity of the sputtered metal is high enough to enable fast charge dissipation.

**Table 3:** Electrode and MCP potentials (with respect to ground potential) during measurement campaigns. For position of the electrodes see Fig. 1

Electrode	Potential [V]	Description
*φ*_1_	255	Backplane
*φ*_2_	20	Ion mirror
*φ*_3_	10	Ion mirror
*φ*_4_	−10	Ion mirror
*φ*_5_	−15	Ion mirror
*φ*_6_	−20	Ion mirror
*φ*_7_	−1190	Drift tube
*φ*_8_	−2050/−300	MCPs (HV/LV)
*φ*_9_	−890	Lens
*φ*_10_	−420	Snorkel
*φ*_11_	−1850	Acceleration

## Appendix

## Author contributions

Salome Gruchola: data curation, formal analysis, methodology, conceptualization, software, visualization, writing – original draft. Andreas Riedo: conceptualization, supervision, project administration, writing – review & editing. Peter Keresztes Schmidt: software, writing – review & editing. Coenraad P. de Koning: writing – review & editing. Luca N. Knecht: writing – review & editing. Marek Tulej: supervision, writing – review & editing. Frances Westall: resources, writing – review & editing. Peter Wurz: supervision, project administration, funding acquisition, writing – review & editing.

## Conflicts of interest

There are no conflicts to declare.

## Supplementary Material
